# Factors Affecting Outcomes of Slipped Capital Femoral Epiphysis

**DOI:** 10.7759/cureus.6883

**Published:** 2020-02-05

**Authors:** Panagiotis V Samelis, Eftychios Papagrigorakis, Apostolos-Lykourgos Konstantinou, Harris Lalos, Panagiotis Koulouvaris

**Affiliations:** 1 Orthopaedics, Children’s General Hospital Panagiotis & Aglaia Kyriakou, Athens, GRC; 2 Orthopaedics, Children's General Hospital Panagiotis & Aglaia Kyriakou, Athens, GRC; 3 Orthopaedics, Katholisches Krankenhaus Dortmund-West - St. Lukas Klinikum, Düsseldorf, DEU; 4 Sports Medicine, Children's General Hospital Panagiotis & Aglaia Kyriakou, Athens, GRC; 5 Orthopaedics, Attikon University Hospital, Athens, GRC

**Keywords:** slipped, capital, femoral, epiphysis, scfe, fai, growth, remodelling, delayed, diagnosis

## Abstract

Slipped capital femoral epiphysis (SCFE) is a frequent cause of nontraumatic painful hip of the adolescence. It is the result of the separation of the proximal femoral growth cartilage at the level of the hypertrophic cell zone. The femoral neck metaphysis rotates externally and migrates proximally relative to the femoral head epiphysis, which is stably seated in the acetabulum; early diagnosis and in situ stabilization grants the best long term results. Numerous factors affect treatment outcomes. Not all implants have the same effect on the slipped physis. Application of the traditionally used implants, such as non-threaded pins and cannulated screws, is questioned. Modern implants are available, which stabilize the slip without accelerating physis fusion. This allows femoral head and neck growth and remodeling to limit the post-slip sequellae on hip anatomy and function. Femoroacetabular impingement (FAI) complicates almost all slips. It causes progressive labral and articular cartilage damage and leads to early hip osteoarthritis and total hip replacement, approximately ten years earlier compared to the general population. Avascular necrosis of the femoral head is a dramatic complication, seen almost exclusively in unstable slips. It develops within months after the slip and leads to immediate articular joint degeneration and the need for total hip replacement. Another serious complication of SCFE is chondrolysis, which is a rapid progressive articular cartilage degeneration leading to a narrow joint space and restriction of hip motion. Implant-related complications, such as migration and loosening, may lead to the progression of the slip. Though bilateral disease is quite frequent, there is no consensus about the need for preventive surgery on the healthy contralateral hip. Diagnosis of SCFE is frequently missed or delayed, leading to slips of higher severity. Silent slippage of the capital femoral epiphysis is highly suspected as an underlying cause of cam-type FAI and early-onset hip osteoarthritis. There is controversy, whether asymptomatic implants should be removed. Novel surgical techniques, such as the modified Dunn procedure and hip arthroscopy, seem to be effective modalities for the prevention of FAI in SCFE.

## Introduction and background

Slipped capital femoral epiphysis (SCFE) is a frequent cause of a nontraumatic painful hip of the adolescence [1,2]. The femoral neck metaphysis rotates externally and migrates anteriorly and proximally relative to the proximal femoral epiphysis, which is stably seated in the acetabulum [2]. Slip stabilization as soon as possible with one cannulated screw is the widely accepted treatment [1-3].

In spite of the simplicity of SCFE pathology and subsequent treatment (“one single screw”!), it seems that this disease bothers the patient for the rest of his life; since long term sequelae or complications of SCFE are not always fully reversible, or may even progress, leading to early-onset disability and need for early hip reconstruction surgery [1,2].

Questions are awaiting an answer:

1. Is the remaining growth of the proximal femur a risk factor for complications or an opportunity to obtain better long term results after SCFE treatment?

2. Is it possible to curtail catastrophic complications, such as avascular necrosis or chondrolysis?

3. Is femoroacetabular impingement a part of the natural history of SCFE or a complication?

4. Is additional surgery, either to prophylactically stabilize a healthy contralateral hip or to remove asymptomatic hardware of the primarily affected hip necessary?

5. Is the mechanism of slippage of the proximal femoral epiphysis a silent ongoing procedure that ultimately results in the degeneration of a previously healthy hip?

6. What is the role of hip arthroscopy or the modified Dunn procedure in the treatment of SCFE?

## Review

1. Implants used for the treatment of slipped capital femoral epiphysis

Slip stabilization is the main goal of any treatment of slipped capital femoral epiphysis (SCFE). This is achieved either with implants routinely used in the orthopedic practice, such as cannulated screws and pins, or implants specifically designed for the treatment of SCFE, such as the telescopic screw and the pin-screw. All implants are effective in stabilizing the slip; however, they have different impacts on the growing potential of the femoral neck growth cartilage.

**Figure 1:**
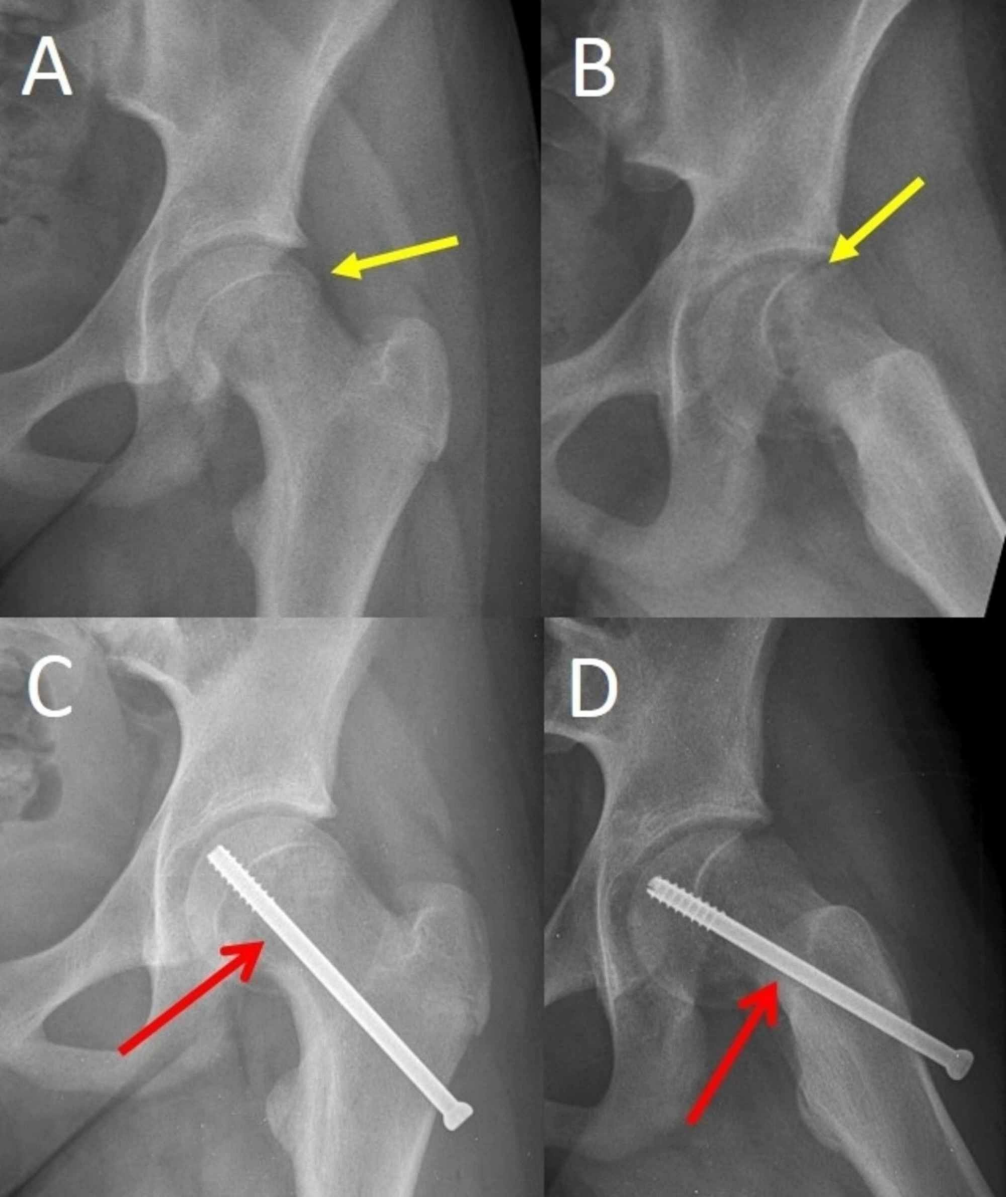
Stable SCFE of the left hip of a 13-year-old girl and in situ stabilization with one cannulated screw (A,B) Anteroposterior and frog-lateral pelvis x-ray scans indicate the slip (yellow arrows). (C,D) Anteroposterior and frog-lateral views of the pelvis indicate in situ stabilization with one cannulated screw (red arrows). SCFE: slipped capital femoral epiphysis

In situ stabilization of the capital femoral epiphysis on the femoral neck metaphysis with one 6-7 mm cannulated screw is the widely accepted treatment for both stable and unstable SCFE (Figure 1) [1]. The cannulated screw effectively stabilizes the physis, with a reported lower risk of complications compared with other implants [1,2]. Furthermore, the typical insertion technique of the screw promotes growth-arrest of the proximal femoral physis and thus limits the risk of slip progression [1].

The screw is inserted under image intensification and, ideally, traverses the center of the capital femoral epiphysis vertically, as seen in the anteroposterior and lateral hip views. Three to five threads of the screw are anchored into the proximal femoral epiphysis to obtain a stable fixation of the slip [2]. The implant is propelled up to 2.5 mm from the subchondral bone of the femoral head [3]. Implants of higher diameter lead to a more stable construct [2].

Intraoperative arthrography or computer navigation may be implemented in order to insert the screw close to the subchondral bone without breaching the articular cartilage [2].

**Figure 2:**
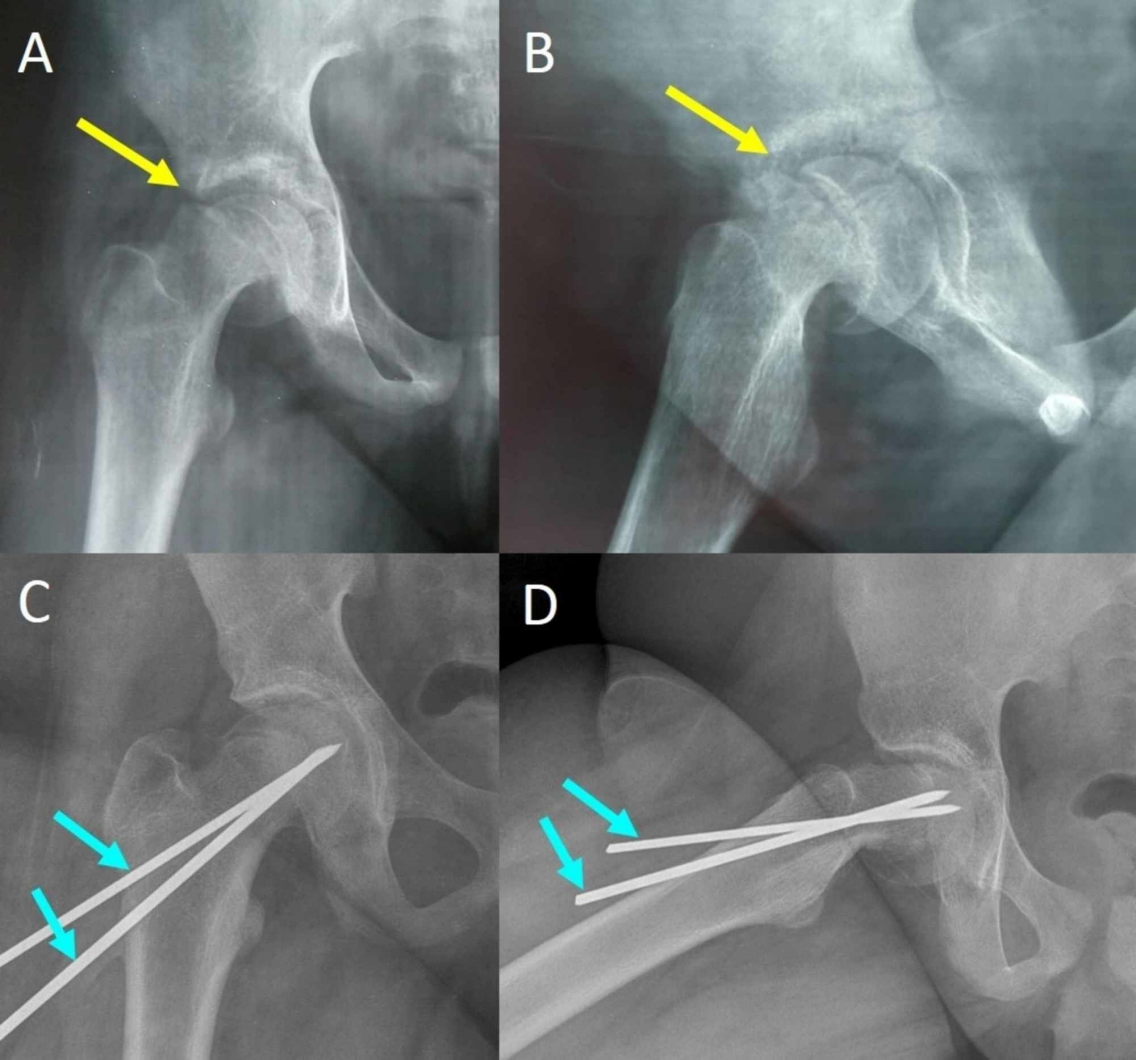
SCFE of the right hip of a 12-year-old girl and in situ stabilization with two smooth pins Anteroposterior (A,C) and frog lateral (B,D) pelvis views indicate the slip (yellow arrows) and the two Steinman pins (blue arrows). SCFE: slipped capital femoral epiphysis

Multiple (two to three) smooth stainless steel pins through the growth plate, driven up to 2 mm from the subchondral bone of the capital femoral epiphysis, are also a safe option to stabilize the slip (Figure 2). Compared to the typical cannulated screw stabilization, multiple pins may spare the remaining growth potential of the femoral neck physis [4,5].

2. Growth-sparing surgery

SCFE affects the growing skeleton. Promoting physeal arrest was the main goal of SCFE treatment in the past in order to avoid slip recurrence due to the continued growth of the femoral neck [1,4]. Recent studies about growth and remodeling of the femoral neck strongly suggest that SCFE treatment should take advantage of the remaining growth of the femoral neck physis, in order to prevent complications such as femoroacetabular impingement and limb length discrepancy [1,4,5]. This is crucial in relatively younger patients [5].

There are two types of SCFE stabilization techniques, according to their effect on the residual growth of the femoral neck physis: growth-restricting and growth-sparing surgical techniques [1].

In the past, most surgeons aimed at physeal arrest, by inserting one cannulated screw in a way, that compresses the capital femoral epiphysis on the femoral neck metaphysis. The thread of the screw bridges the physis while the base of the screw is in contact with the lateral cortex of the femur. Studies show that this technique leads within 6-12 months to physeal arrest [4,6,7]. However, early physis closure eliminates any chance of the hip to improve the SCFE-related femoral neck deformity [1,4].

There are various techniques to stabilize the slip without restricting the residual growth of the physis: 

2.a. Non-Threaded (smooth) Pins

Percutaneously inserted 2-3 smooth pins (5-6mm) across the femoral neck growth plate are effective in stabilizing the slip without accelerating physis fusion [1,3].

2.b. The Gliding Screw Principle

A special surgical technique may allow the typical cannulated screw to stabilize the physis without promoting early fusion. The tip of the screw has only a few threads that are contained in the proximal femoral epiphysis. The shaft of the screw is long enough to protrude 1.5-3 cm out of the lateral cortex of the femur. For an expected residual growth of 2-3 years, the screw, firmly anchored in the capital femoral epiphysis, glides into the growing femoral neck, until the head of the screw abuts the lateral femoral cortex. At this point, the screw stops gliding, and with further growth, the screw practically compresses the physis. If further growth is expected, the screw has to be replaced by a longer one - based on the same principle of gliding - to resume growth-preserving stabilization [3].

Growth-sparing stabilization using the gliding screw principle or stabilization with stainless non-threaded pins leads to simultaneous fusion of the treated and the healthy contralateral hip, within 31-37 months [3,4,8]. Moreover, the articulotrochanteric distance (ATD: distance of the roof of the femoral head and the tip of the greater trochanter) between the affected and the prophylactically treated hips, is comparable, indicating continuing femoral neck growth after treatment [4]. Other studies showed that growth-preserving stabilization of the SCFE and the healthy contralateral hip leads to simultaneous physis fusion of both hips (at about 17 months postoperatively). The healthy contralateral hips present more prominent growth, inferring that some injury of the affected growth plate may be permanent, or the slipped physis is primarily deficient [4,8,9].

2.c. The Pin-Screw

The thread of the pin-screw is located at its base. The tip of the screw is non-threaded and enters the capital femoral epiphysis, up to 2 mm from the subchondral bone [4]. Femoral neck growth forces the capital femoral epiphysis to slide along the screw. Thus the pin-screw allows the femoral neck to grow, leading to a longer femoral neck and a higher head-neck offset. However, the changes of the affected hips are less pronounced compared to the healthy contralateral hips that were treated with the same implant, indicating a primarily deficient or irreversibly injured femoral neck physis. The pin-screw provides adequate stabilization of unstable slips as well [4].

2.d. The Telescopic Screw

The Telescopic Screw consists of two cylindrical parts, one contained in the other [10]. The epiphyseal part is of smaller diameter and has a thread, which is completely driven into the epiphysis. The metaphyseal part is of greater diameter and is threaded at its base, which is driven into the lateral cortex of the femur and the base of the femoral neck. Like a telescope, the epiphyseal part glides in the metaphyseal part. Ongoing growth of the femoral neck forces the epiphyseal part of the screw to glide out of the metaphyseal part, as it follows the moving capital femoral epiphysis. A study has shown a decrease in the slip angle by 11⁰ and the alpha-angle by about 30⁰ after stabilization of mild and moderate slips with this screw [10]. Most correction (about 60%) was observed during the first postoperative year, stressing the significance of early diagnosis in order to exploit as much as possible of the remaining growth and remodeling potential of the hip. The Telescopic Screw may be applied in unstable SCFE as well. In this case, an additional smooth pin should be added to provide rotational stability [10].

3. Complications of SCFE

3.a. Avascular Necrosis of Femoral Head

Avascular necrosis of the femoral head (AVN) is the most serious complication of SCFE, observed almost always after unstable slips (up to 47% of the cases) [1,11]. AVN may develop within a few months after the slip. Hip pain and limp worsen with time. AVN usually affects the anterosuperior portion of the femoral head [11]. Symptoms exacerbate with the collapse of the necrotic bone, after which rapid destruction of the joint is inevitable. Bone scintigraphy is useful to evaluate femoral head viability after the stabilization of unstable SCFE [12].

**Figure 3:**
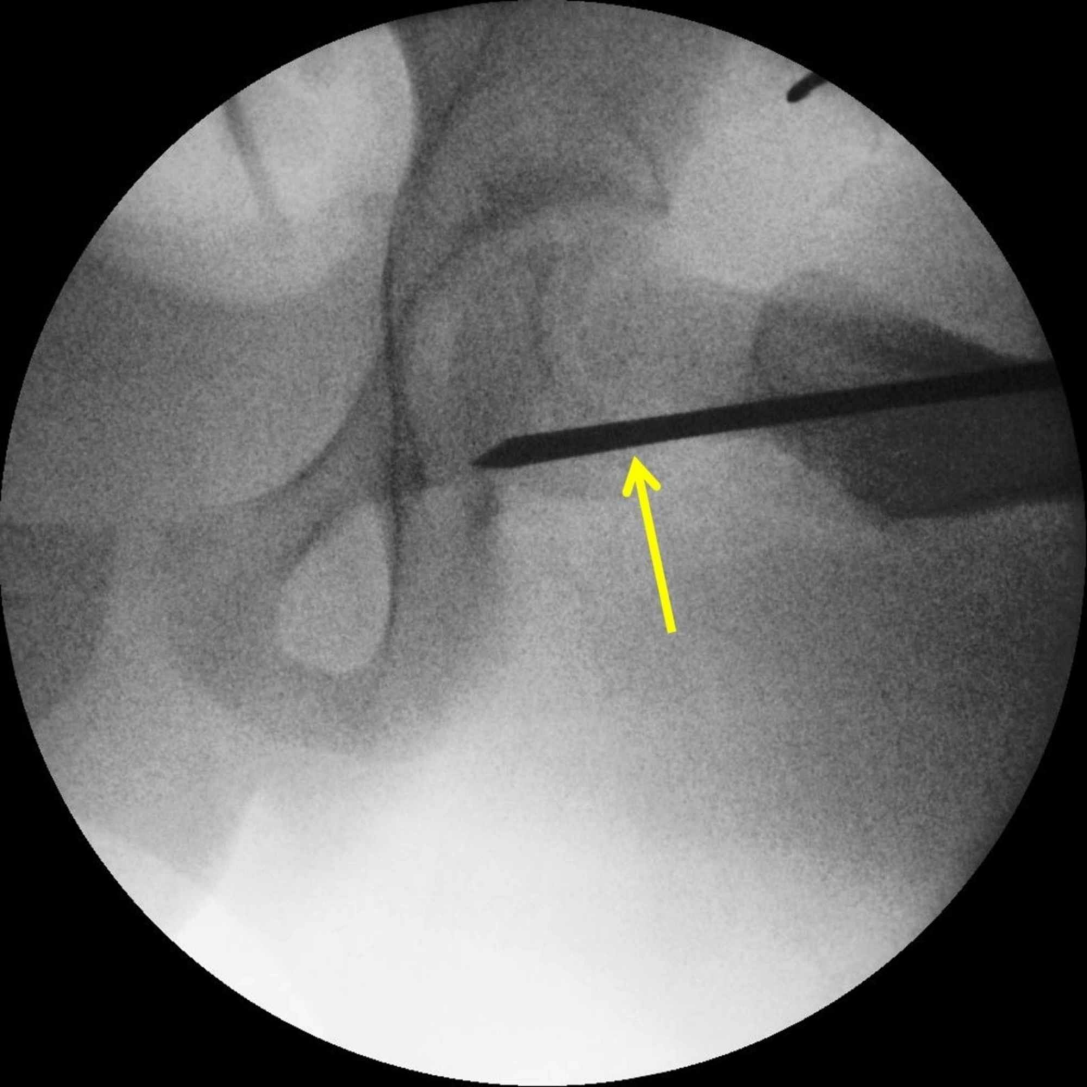
Potential injury of the nutrient vessels of the femoral head by a posteriorly inserted pin, the pin exits the femoral neck and enters the posterosuperior portion of the femoral head Arrow indicates the posterior track of the implant relative to the femoral neck

Unstable SCFE is the main risk factor for AVN; however, AVN may be iatrogenic, as in case of injury of the nutrient vessels by a posteriorly inserted pin or screw, that exits the femoral neck and enters the posterosuperior aspect of the epiphysis (Figure 3). Overzealous efforts to reduce an unstable slip anatomically or any reduction of a stable slip will compress the nutrient vessels on the neck callus and lead to AVN. Femoral neck osteotomies to prevent post-slip neck deformity may also jeopardize the femoral head arterial supply [7,13].

The nutrient vessels of the proximal femoral epiphysis may be injured or obstructed, either at slip completion or secondary to slip reduction [14]. Increasing evidence supports that anatomical reduction of an unstable slip by closed means should be avoided. Attempts at closed reduction may raise the intraarticular pressure of the hip joint to levels above the pressure of compartment syndrome and may, therefore, harm the vascular supply of the femoral head [14]. Furthermore, the treatment of unstable SCFE using the modified Dunn procedure has shown that almost all unstable slips present signs of preceding chronic disease (posteroinferior neck callus). Any attempt to reduce an unstable slip anatomically will press the retinacular vessels on this callus. Consequently, closed anatomical reduction of unstable SCFE should be avoided in favor of incidental reduction [11,14]. Anatomic reduction of the femoral head epiphysis in unstable SCFE should be tried only by open surgery, after callus removal and femoral neck shortening [7,15].

3.b. Chondrolysis of the Hip Joint

Slip stabilization leads to rapid resolution of hip pain. Unremitting symptoms after surgery may be the result of chondrolysis, which is defined as a progressive articular cartilage absorption [1]. Instead of improving after surgery, the clinical presentation of the patient deteriorates. The patient complains of increasing hip pain, restriction of motion, and limp [7]. Pain may reflect on the thigh or knee. A reduction of the intraarticular space by more than 50% of the asymptomatic contralateral hip, as seen on the pelvis x-ray, is diagnostic of chondrolysis. In the case of bilateral SCFE, a joint space less than 3mm in the presence of ongoing symptoms is considered indicative of chondrolysis [1,7].

**Figure 4:**
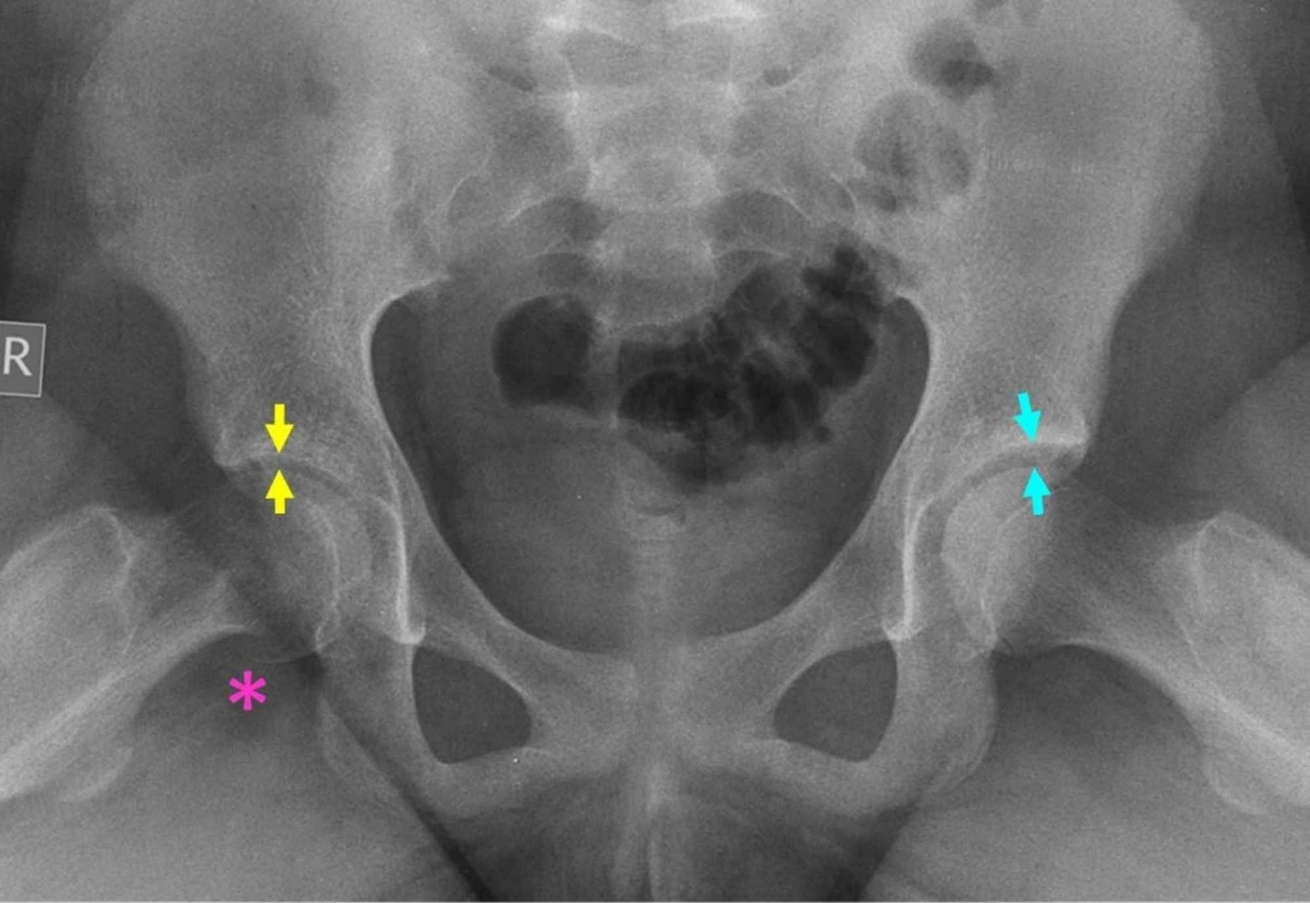
Chondrolysis secondary to delayed treatment of SCFE of the right hip in a 12-year-old girl The joint space of the right hip (yellow arrows) is less than 50% of the joint space of the left hip (blue arrows). The asterisk indicates the posteroinferior callus of the femoral head-neck junction. SCFE: slipped capital femoral epiphysis

The frequency of chondrolysis is 5-7% [1]. Its etiology is unclear. Factors that have been associated with increased risk for chondrolysis are autoimmune factors, prominent hardware into the joint space, hip-spica treatment, severe SCFE, obese patient, delayed diagnosis, and subtrochanteric osteotomy before growth plate fusion (Figure 4) [1,7]. Temporary protrusion of the implant into the joint space during slip stabilization does not cause chondrolysis [7,16]. Treatment includes removal of the protruding implant, physical therapy, and analgesics. Effective treatment leads to restoration of the joint space and remission of symptoms within 10 months [7]. 

3.c. Femoroacetabular Impingement and Early Hip Degeneration

Femoroacetabular impingement (FAI) is observed in almost all cases of SCFE [1,2,6-8,10,15,17-21]. Mild slips are not spared from this complication. FAI will almost always lead to labral tears and articular cartilage injury of the acetabulum [1,17,18,19,20]. FAI could actually be deemed not a complication, but the endpoint of the natural history of SCFE, whether untreated or after in situ stabilization [1]. After this point, secondary disease and reconstruction surgery of the hip will probably not be avoided [1].

FAI occurs during flexion and internal rotation of the SCFE hip when the deformed femoral neck abuts against the acetabular labrum and the acetabular articular cartilage. Two types of SCFE-related FAI have been described. The first type is seen in mild and moderate slips when the deformed femoral neck enters the joint and causes abrasion of the anterosuperior labrum and the articular cartilage of the acetabulum (cam-type or inclusion-type FAI) [1,22]. In severe slips, the deformed femoral neck can no longer enter the acetabulum, but it impacts the rim of the acetabulum (pincer type, impaction type impingement) [22]. Severe slips present both lesions of the acetabular rim and intra-articular lesions as well. The latter is suggested to be the result of impaction-type impingement that occurred at the early stages of the slip [1,23].

Remission of hip pain and improvement of the limp is expected soon after slip stabilization [1,21]. Depending on slip severity, FAI becomes symptomatic months or years after surgery due to permanent labral and/or articular cartilage injury [1,15]. FAI is strongly suspected in any post-slip hip with restricted internal rotation (<10⁰) in 90° of flexion or inability of flexion beyond 90° [19]. Flexion, adduction, and internal rotation of the hip (positive FADIR sign) elicits hip pain [21]. Hip flexion leads to progressive external rotation of the thigh (positive Drehman sign), as the patient involuntarily tries to avoid the impaction of the anterosuperior femoral neck on the anterosuperior acetabulum [1,21,22].

Slip severity correlates with the risk for FAI [1]. FAI complicates 100% of severe slips, 50% of moderate slips, and 33% of mild slips [24]. Mild slips are not free of risk for FAI [7,15,24,25]. In the long term, regardless of slip severity, 80-90% of the treated slips will present labral and articular cartilage lesions of the acetabulum [1]. Labral lesions appear within 6-12 months after the slip onset and are located between the 10th and the 3rd hour of the acetabulum [15,26,27]. Later on (within about three years), articular cartilage defects appear [1,26]. The labral and cartilage damage may be asymptomatic for a long time before the hip becomes painful. After this point, the progressive degenerative hip disease will lead to early reconstructive hip surgery [15,27]. Interestingly, slips that were found to be unstable during open surgery (modified Dunn procedure) presented less labral and acetabular cartilage damage compared to chronic stable hips. One possible explanation is that the intense clinical presentation of an unstable slip forces the patient to seek early medical care before FAI-induced lesions appear [1,15].

**Figure 5:**
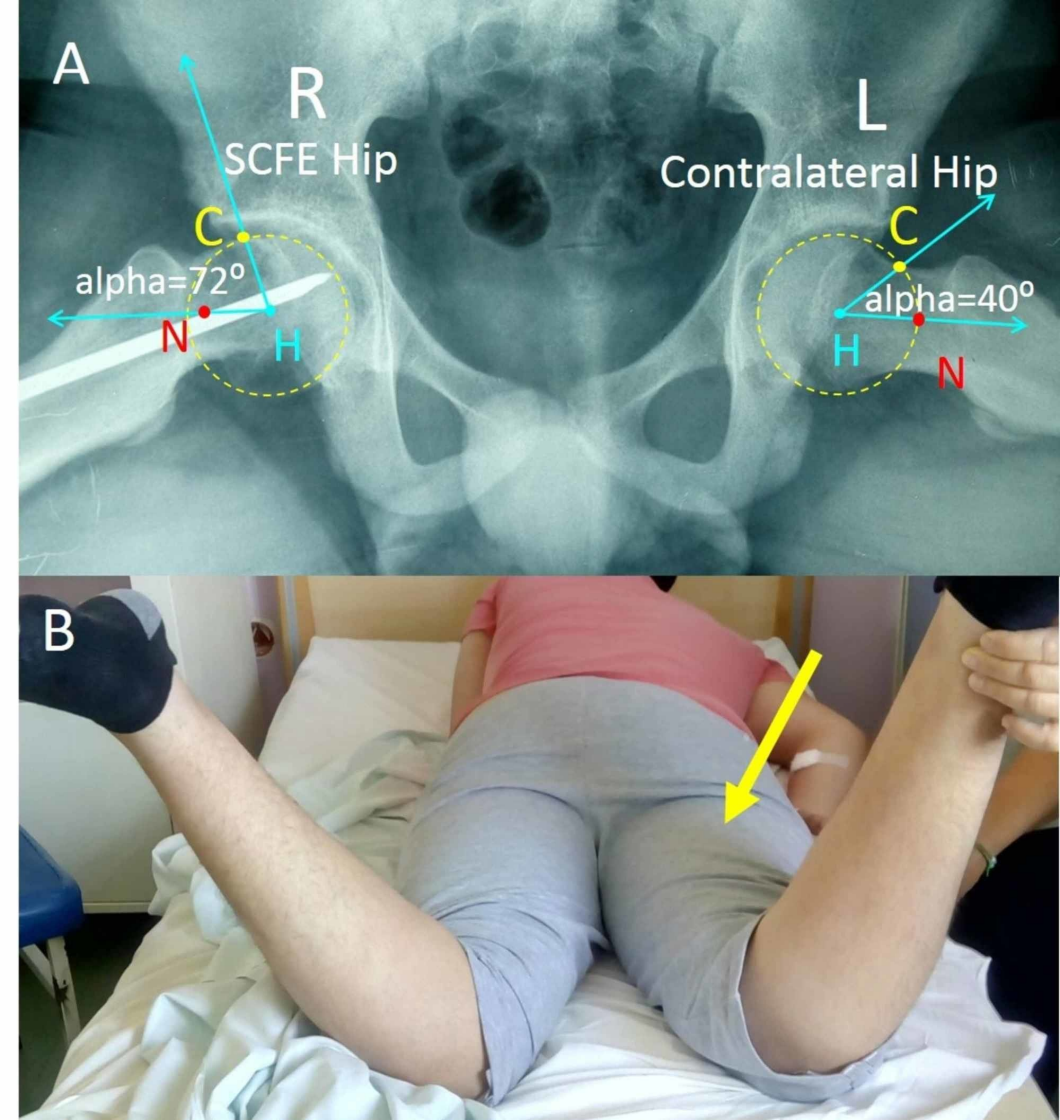
SCFE of the right hip of a 14-year-old boy (A) Measurement of the alpha-angle on the frog-lateral pelvis projection. The alpha-angle is formed between the lines HC and HN. Normally, the alpha-angle is less than 55 degrees. (B) Restricted internal rotation of the right hip of the patient indicating FAI (arrow) SCFE: slipped capital femoral epiphysis; FAI: femoroacetabular impingement; H: femoral head center; N: center of the narrowest point of femoral neck; C: the point where the femoral head radius intersects the continuation of the femoral neck; R: right; L: left

**Figure 6:**
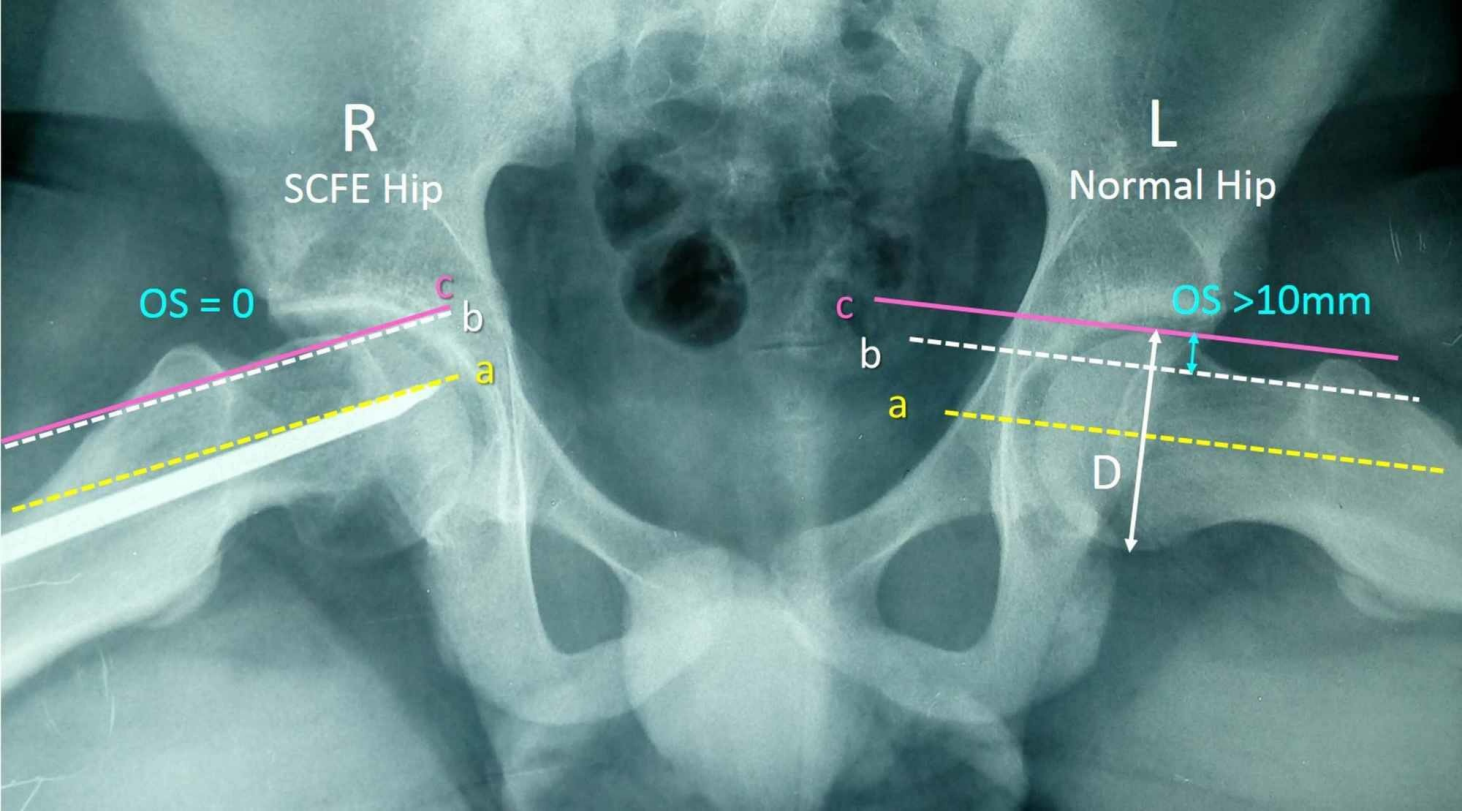
Measurement of the anterior head-neck offset (OS) and the anterior head-neck offset ratio (HNOR) on the Lauenstein pelvis projection of the patient in Figure 5 OS is the distance between the lines c and b and is normally ≥10 mm. HNOR is the ratio OS/D and is normally >0.15. Right hip shows normal OS and HNOR but the left hip has post-SCFE FAI with pathologic OS and HNOR. SCFE: slipped capital femoral epiphysis; FAI: femoroacetabular impingement; a: axis of the femoral head; b: line on deepest point of the anterior surface of the femoral neck, parallel to a; c: line on the peak of the anterior contour of the femoral head, parallel to a; D: diameter of femoral head; R: right; L: left

The frog-lateral (Lauenstein) pelvis projection is used for the radiologic diagnosis of FAI (Figures 5, 6) [28]. The alpha-angle (normally <55°), the anterior head-neck offset ratio (HNOR: neck-head offset divided by the femoral head width, normally >0.15), and the anterior femoral head-neck offset (OS, normally >10mm) are useful radiologic measurements to describe an abnormal femoral head-neck junction and to establish the diagnosis of FAI on a painful post-slip hip [28]. Other x-ray views, which may be used for the radiologic assessment of FAI-associated femoral neck deformity, are the 45° Dunn view (45° hip flexion, neutral rotation, 20° abduction) [21]. The alpha angles of the SCFE hip and the opposite asymptomatic hip should be compared in order to diagnose symptomatic FAI of the SCFE hip [1].

On the contrary, radiologic signs of SCFE and post-slip radiologic signs indicating FAI (slip angle, alpha angle), do not always correlate with the severity of the clinical presentation of FAI [21]. This is because the development of symptomatic FAI depends on factors such as the patient's occupation and level of physical activity, and other anatomic factors of the hip as well, such as the hip offset, the acetabular depth (coxa profunda) and the inclination of the femoral neck and the acetabulum (acetabular retroversion is associated with both types of FAI) [1,7,21,24]. All these factors participate in FAI pathology and clinical presentation.

**Figure 7:**
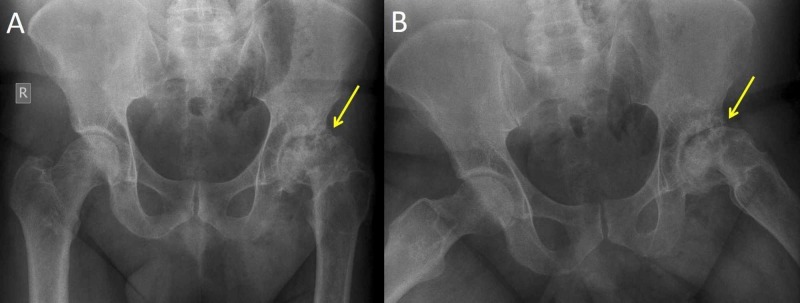
Marked osteoarthritis of the left hip secondary to non-diagnosed SCFE in a 40-year-old man The x-ray was taken while his 11 year-old-daughter was treated for bilateral SCFE. Anteroposterior (A) and frog-lateral (B) pelvis views indicate marked osteoarthritis secondary to untreated SCFE of the left hip (arrows). SCFE: slipped capital femoral epiphysis

The frequency of SCFE in the total number of total hip replacement (THR) is relatively low. Among 370,630 primary total hip arthroplasties (THAs) reported from the Nordic Arthroplasty Register Association for 1995-2009, SCFE and Perthes' disease as a group was reported to be responsible for only 0.6% of primary THRs [29]. However, the patients of the SCFE/Perthes group were significantly younger (mean age, 49.7 years) compared to the patients with primary hip osteoarthritis (69.3 years) [29]. Overall, post-slip FAI leads to early hip osteoarthritis and total hip replacement (THR) at a younger age (Figure 7) [25]. It seems that SCFE patients will undergo a THR approximately 10-12 years earlier than patients with idiopathic hip osteoarthritis [17,20,25]. Radiologic signs indicating a history of SCFE are observed 35.7% of patients with hip osteoarthritis younger than 60 years of age [20]. Other studies support that SCFE is the underlying cause in up to 6,5% of hip osteoarthritis and in 9.3% of THR in patients younger than 50 years [30,31].

3.d. Implant-Related Complications

Treatment for SCFE bears complications that are related to the surgical technique and the type of implant that is used [1]. Non-threaded pins may migrate, thus leading to slip recurrence [16]. Thin pins may bend. The implant may exit the femoral neck and enter the posterosuperior portion of the femoral head. At this point, the nutrient vessels of the epiphysis may be harmed (Figure 3) [13]. A prominent implant into the joint space will cause chondrolysis [7,16]. Continuing femoral neck growth may cause the epiphysis to uncouple from a non-threaded pin and slip further on the femoral neck [8]. Repeat loading on the prominent end of a pin that projects out of the lateral femoral cortex may result in pin loosening and migration with subsequent recurrence of the slip [8]. A prominence greater than 1.5 cm may exert a "windshield wiper" loosening effect on the implant, which is caused by the forces exerted on the implant from the overlying soft tissues [16]. Repeat drilling of the lateral femoral cortex during implant insertion may predispose to a pertrochanteric fracture of the femur [8,13]. Insertion of the implant at or below the lesser trochanter increases the risk of a subtrochanteric fracture as well. Multiple implants are associated with a higher risk of complications [16].

4. Simultaneous stabilization of the asymptomatic contralateral hip

**Figure 8:**
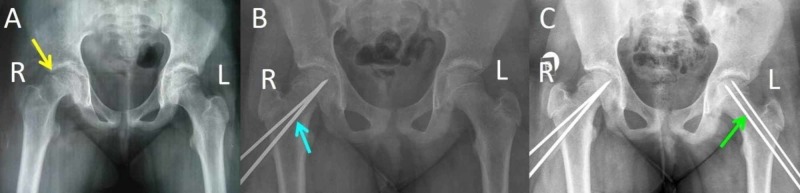
Contralateral (Left) SCFE 10 months after primary SCFE of the right hip, in the patient shown in Figure 2 (A) SCFE of the right (R) hip (yellow arrow), (B) in situ stabilization of the R hip using two Steinman pins (blue arrow), (C) in situ stabilization of the left (L) hip with two Steinman pins (green arrow) SCFE: slipped capital femoral epiphysis

The incidence of bilateral SCFE is quite frequent. Most reports agree with an assessment of 50% bilateral hip disease within two years of the primary hip SCFE [1,16,32]. The risk of a contralateral slip increases dramatically (up to 100%) in obese patients or patients with an underlying endocrine disease [33,34]. Contralateral SCFE may be observed at the same time with the index hip (8-27%), or later, usually within 3-5 months (19-40%) (Figure 8) [8,33]. The contralateral slip may be symptomatic (pain, limp). Occasionally, diagnosis of the contralateral hip disease is made by chance (typical musculoskeletal survey after trauma) without preceding symptoms. In fact, most cases of contralateral SCFE (41-92%) are silent, and when detected in the adult, they frequently (29%) present secondary degenerative joint disease [1,32,33].

Simultaneous contralateral hip treatment is still a controversy among orthopedics. The contralateral hip will always be treated if symptomatic (pain, limp), even without radiologic evidence of a slip or a pre-slip. It should also be operated if it is asymptomatic, but with an x-ray of a slip or a pre-slip (wide physis). There is no consensus whether the clinically and radiologically normal contralateral hip should receive preventive stabilization, simultaneously with the symptomatic index hip, in order to avoid a future contralateral slip [33,34].

Studies against simultaneous preventive surgery of the painless contralateral hip state, that this additional procedure may present complications such as AVN and chondrolysis as well [1,7,16]. Other studies support that in spite of an increased alpha-angle, frequently observed on the asymptomatic contralateral hips of SCFE patients, it is not certain that these hips are at increased risk for FAI and secondary osteoarthritis [17]. Furthermore, prophylactic stabilization of a healthy contralateral hip may, in fact, be unnecessary surgery because the contralateral SCFE is usually a mild, acute slip (73-78%), that is timely diagnosed (first hip history) and treated [1,35]. Besides, it is not certain that preventive physis stabilization will protect the contralateral hip from developing slip-like morphology of the femoral neck, such as a pistol grip deformity or femoral head retroversion [35]. Slip-like morphology is incidentally observed in healthy adults, and it is not certain that it leads to FAI and degenerative joint disease [35].

**Figure 9:**
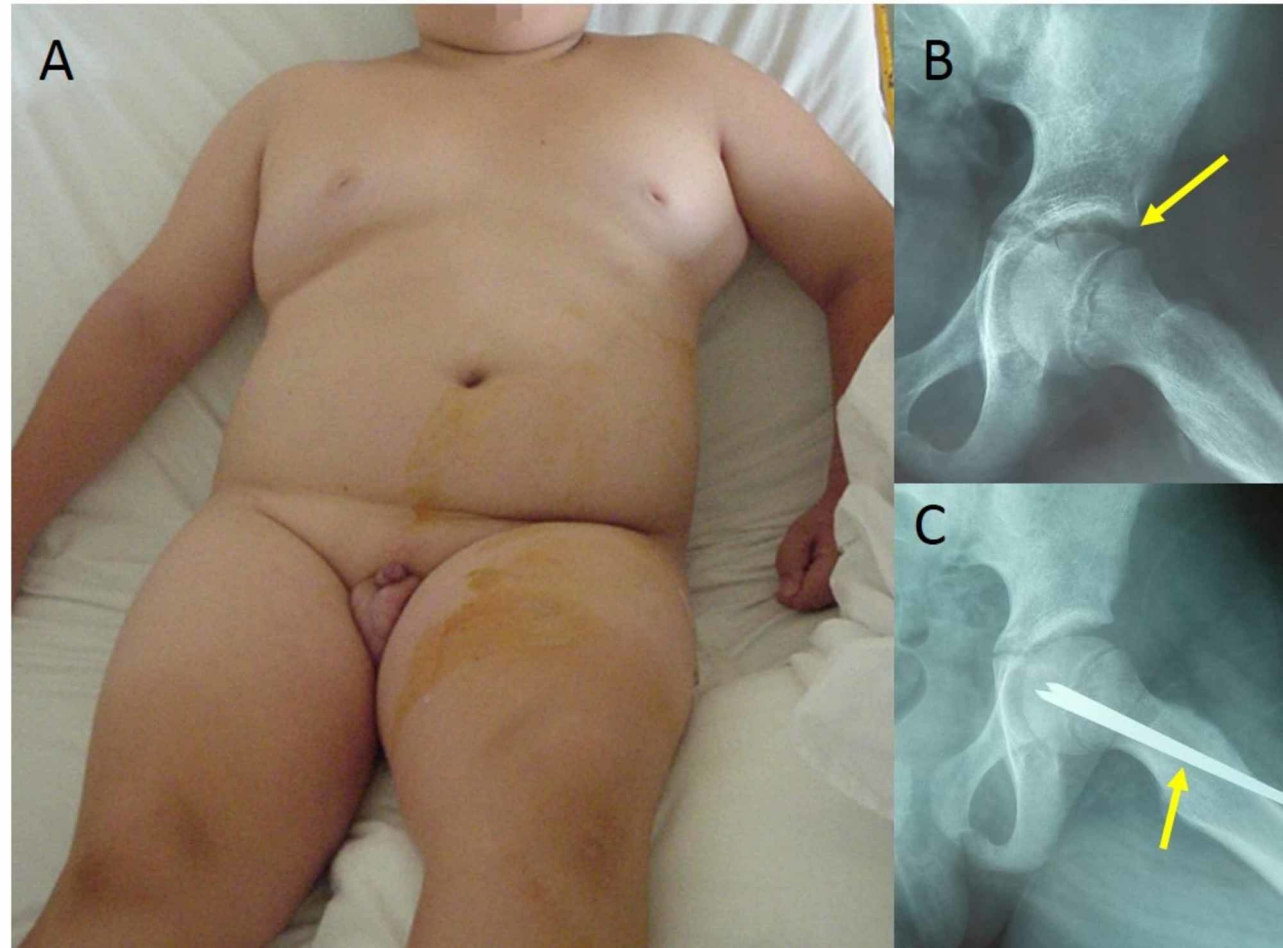
SCFE of the left hip in a 9-year-old obese boy with hypotrophic external genital organs (A) the adiposogenital phenotype: obesity combined with small external genital organs, (B) frog-lateral x-ray of the left hip indicates the SCFE (arrow), (C) in situ stabilization with two 6 mm Steinman pins (arrow) SCFE: slipped capital femoral epiphysis

Studies that favor simultaneous surgery of the asymptomatic contralateral hip support the rationale that preventive physis stabilization has low perioperative morbidity and complications compared with therapeutic SCFE surgery [10]. Other studies report that preventive physis surgery may prevent a silent slip and radiologic evidence of Cam-type FAI [21]. Nevertheless, simultaneous prophylactic contralateral physis stabilization should be a serious option in the case of a younger patient, an underlying endocrine disorder, the adiposogenital phenotype of the patient (Figure 9), and in case social or geographical factors may hinder the patient from seeking immediate medical help [1]. The same surgical technique should be used on both hips in order to impose the same effect on the remaining growth on both hips [1].

Efforts have been made to predict the risk of contralateral hip SCFE, in order to select patients with for targeted preventive contralateral physis stabilization [1].

The posterior sloping angle (PSA) of the femoral neck is calculated on the frog lateral pelvis view. It is the angle between the line of the physis and a line vertical on the femoral neck-shaft axis. The PSA differs between SCFE and healthy hips [7]. Studies suggest that a PSA greater than 12⁰-15⁰ should be an indication for prophylactic surgery on the contralateral hip [7,34].

The modified Oxford score assesses the risk of contralateral SCFE by scoring five radiologic factors on the anteroposterior pelvis projection: Stages of maturation of the iliac crest, the triradiate cartilage of the acetabulum, the proximal femoral epiphysis, the trochanter major and the trochanter minor receive a score. A lower total score indicates a more immature patient with a higher risk of future contralateral SCFE [34].

The maturation of the triradiate cartilage of the acetabulum is useful as an independent prognostic factor for increased contralateral SCFE risk. Wide-Open triradiate cartilage has a probability of 89% for future contralateral SCFE [34].

The alpha angle has also been studied as an independent prognostic factor for future contralateral hip disease: an alpha angle >50.5⁰ implies a higher risk of impending contralateral SCFE and is suggested as a cut-off point for contralateral preventive surgery [34,36].

Early intervention on the asymptomatic contralateral hip should always be considered in the presence of obesity (Bone Mass Index - BMI >95th percentile, >35kg/m2), young age (less than 10 years for girls, less than 12 years for boys), female patient and underlying hormonal disease [1,7,34,37].

5. SCFE pathology contribution to primary hip osteoarthritis

5.a. Silent SCFE

Hip pathology indicating a previous slip has been frequently reported in adults. Radiological findings resembling a prior silent SCFE were found in 6.6% of a cohort of 2072 healthy young adults [38]. A retrospective study supports that 24.7% of adults who had a THR for coxarthrosis present radiographic signs of SCFE [24]. Another retrospective study found that 12% of hips with a primary cam deformity presented a slip-like morphology [39]. Moreover, most contralateral slips are first diagnosed in adulthood without a positive history of the hip disease [32].

It has been suggested that this frequent finding of SCFE morphology in hip osteoarthritis may be the result of a subclinical (silent or asymptomatic) SCFE, which stops with growth plate closure. Beyond some point, and depending on other patient-related factors, these silent slips cause symptoms of FAI in the adult. A slip angle greater than 13⁰ at growth plate fusion without a history of hip pathology (pain, limp) when the patient was an adolescent, confirms the diagnosis of an asymptomatic SCFE [35]. 

5.b. FAI-associated Femoral Neck Deformity: Post-Slip vs. Slip-Like

The origin of proximal femoral deformity, which is associated with cam-type FAI, is controversial. It is certain that SCFE leads to proximal femoral deformity, which is termed pistol-grip deformity and leads to cam-type FAI. However, not all cam-type FAIs are the result of SCFE.

Decades ago, Murray described the "tilt deformity of the femoral head", which is a deformity of the proximal femur similar to the pistol grip deformity observed after SCFE. Murray examined the radiographs of 200 patients with primary osteoarthritis (no history of hip disease during childhood) and found that the "tilt deformity of the femoral head" was present in 39.5% of cases [30]. This deformity affects men predominantly. The tilt deformity leads to symptoms (hip pain, limp) before hip osteoarthritis is evident on the x-ray, suggesting that a period of FAI precedes irreversible acetabular labrum and cartilage injury. The author presumes that the tilt deformity of the femoral head is the result of minor trauma during adolescence [30]. 

Whether the tilt deformity of the femoral head is the result of an undiagnosed SCFE is not clear. A positive fovea sign (the neck axis does not intersect the fovea capitis) and a tilt-angle of the femoral head (formed by the perpendicular to the base of the capital femoral epiphysis and the neck axis) greater than 4⁰ are suggested to define a slip-like deformity, similar to the deformity observed after known SCFE [39]. Among 236 hips with cam-type FAI, 12% were deemed slip-like (negative SCFE history) and 3% post-slip (after treated/diagnosed SCFE) [39].

6. Delayed or missed diagnosis of SCFE

Delay or even loss of diagnosis of SCFE is probably the most important factor that affects the long-term outcomes of SCFE [17,33,38,40,41]. Recent studies report an average delay in the diagnosis of SCFE of about 5-7 months, with more than three years being the most extreme reported delay [1,17,40,41].

Several factors lead to delayed diagnosis of SCFE. The patient and his family may neglect minor hip pain or limping and never seek medical advice. Geographical factors may be an obstacle to easy access to any health care system. Unfortunately, in almost half of the cases, the cause of delayed diagnosis is the physician, who first examines the nontraumatic, limping, obese adolescent. In this case, the delayed diagnosis is a missed diagnosis [1].

SCFE is a surgical emergency. Nevertheless, very often, the clinical presentation may be mild, such as in case of a stable, slowly evolving slip, which presents relatively mild symptoms. Location of pain may mislead the physician: only 50% of patients locate the pain at the hip [40]. Pain may reflect on the ipsilateral knee (26%), or thigh (16%), or the patient may report just a painless limp (8%) [40]. Not infrequently, the doctor requests an x-ray of the thigh or the knee [1]. Clinical examination and suspicion of hip pathology are of utmost importance in the limping adolescent. Furthermore, the physician should be aware that the classic anteroposterior pelvis view has low sensitivity for an SCFE diagnosis, missing almost all mild slips [1]. The frog-lateral pelvis projection is the examination of choice for establishing the diagnosis of SCFE. Unfortunately, this projection is either ignored or not ordered on patient admission, in an attempt to spare the patient from additional radiation exposure [1,42]. Repeated admissions and examinations of the limping patient will usually lead to diagnosis and surgery, at the expense of a slip of higher severity and worse long-term results after treatment [1,17,33,38,40]. Slip severity increases by one level for each month of delay of the diagnosis [1,17].

Bearing in mind that 94-96% of mild slips (slip-angle <30⁰) have favorable long term outcomes, that the remaining growth and remodeling of the femoral neck will decrease the slip-angle by 10⁰-15⁰ and the alpha-angle by 10⁰-30⁰, and that FAI is usually associated with a slip-angle >30⁰ and an alpha-angle >55°, it is inferred that a delayed/missed diagnosis spares the hip the opportunity to correct a minor post-slip deformity and thus to avoid FAI [1]. Since in-situ stabilization is the universally accepted surgery for all slips, it seems that early diagnosis is to date the most important factor to improve the prognosis of SCFE after in situ stabilization [1,40,41]. 

7. Growth and remodeling after treatment of SCFE

7a. The Remaining Growth of the Hip After Slip-Stabilization

SCFE is a disease of the growing skeleton. It is expected that the remaining growth of the hip will be affected, either primarily, by the process that caused the slip, or secondarily, by the surgical technique or by the delay of treatment [3-10].

**Figure 10:**
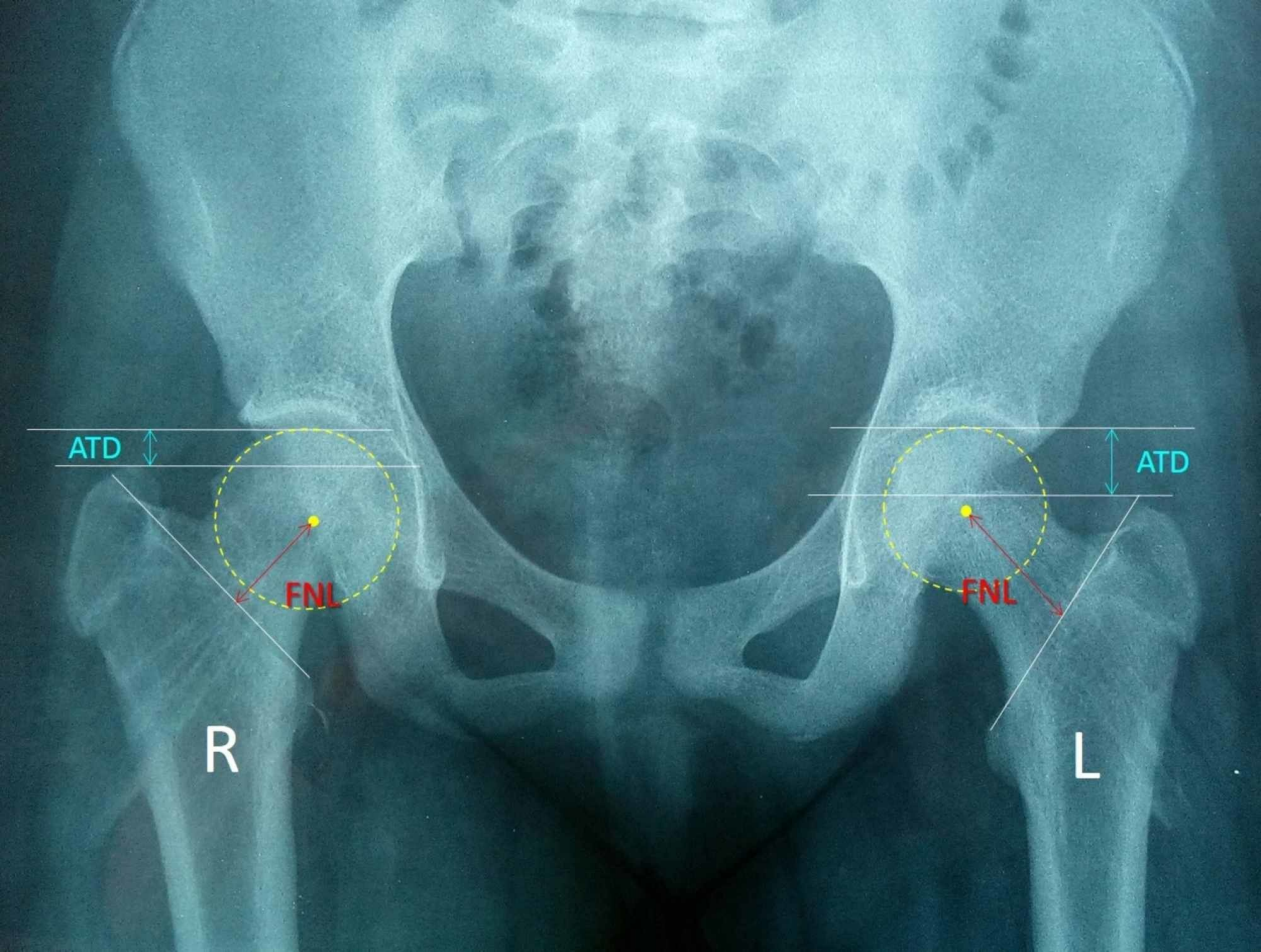
The anteroposterior pelvis projection of the patient of Figure 2 after implant removal shows obvious growth disturbance of the right hip with a shorter ATD and FNL compared to the left prophylactically-pinned hip R: right, L: left, ATD: articulotrochanteric distance, FNL: femoral neck length

Growth disturbance of the hip after SCFE is best assessed on the anteroposterior pelvis view (Figure 10). The articulotrochanteric distance (ATD: the distance between the tip of the greater trochanter and the top of the femoral head) and the femoral neck length (FNL: the distance between the center of the femoral head and the intertrochanteric line) are useful measurements of the growth of the hips after SCFE.

7.b. Remodeling of the Femoral Head-Neck Junction

The femoral neck remodeling of the SCFE hip consists of bone absorption at the anterosuperior surface of the femoral neck metaphysis and bone deposition at the posteroinferior aspect of the metaphysis. Femoral neck remodeling starts shortly after the slip initiation [1,43]. Callus formation at the posteroinferior head-neck junction is detected on ultrasound three weeks after the slip onset and hallmarks the transition of the acute slip to a chronic one [1,43].

Bone remodeling of the femoral head-neck junction is evident in the frog-lateral pelvis projection [1]. Bone absorption at the anterosuperior aspect of the femoral neck metaphysis results in "rounding up" of the respective portion of the femoral neck. On the posteroinferior aspect of the femoral neck metaphysis, the newly formed callus is ossified [1]. The femoral neck is shorter and thicker compared to the healthy contralateral hip. The femoral neck resembles the grip of a pistol [1,6,9,20-22,24,28,31,38,39].

The remaining growth and remodeling potential may improve the post-slip deformity of the hip (Table 1) and thus prevent FAI or gait disturbance; however, this process is not unrestricted [6,8,10,44,45]. Limited improvement of FAI-related parameters (slip-angle, alpha-angle, head-neck offset) is expected, especially in younger patients [6]. Studies have shown that the growth and remodeling potential after SCFE are insufficient to reverse a slip-angle greater than 30⁰-40⁰ and thus will not be able to prevent FAI [6,24]. Thus a slip-angle >30⁰ might be an indication for additional surgery to prevent FAI (arthroscopic osteochondroplasty, open osteochondroplasty, modified Dunn procedure) [1,24].

**Table 1:** The correction of FAI-associated parameters due to growth and remodeling after SCFE SCFE: slipped capital femoral epiphysis, FAI: femoroacetabular impingement, HNOR: head-neck offset ratio

Study	Growth and Remodeling of the SCFE hip
Jones et al. 1990 [6]	- Retrospective study, 70 hips, 7.1 years after in situ pinning
	- Remodeling observed in 90% of mild and 50% of moderate SCFE
	- Sufficient remodeling with Southwick angle ≤40⁰
	- Effect of growth and remodeling on hip motion: increase of internal rotation of the hip.
Kumm et al. 2001 [8]	- Retrospective study, 29 hips, slip-angle <30 degrees, gliding cannulated screw
	- Increase of femoral neck length: 15-30mm,
	- Southwick angle reduction: 15%
Akiyama et al. 2013 [44]	- Retrospective study, 69 hips, in situ pinning
	- Alpha-angle correction: 24,9⁰
	- HNOR correction: 0.086 => 0.135 (normal: above 0.15)
	- Residual neck deformity in 29.4 % of patients
Schumann et al. 2016 [10]	- Retrospective study, 19 cases, stable and unstable SCFEs, Telescopic screw fixation
	- Slip angle decrease: 11⁰
	- Alpha-angle correction: 29.3⁰
	- Correction of the neck varus: 82% of patients
	- Maximal correction obtained within 6-12 months after surgery
Örtegren et al. 2018 [45]	- Retrospective study, 54 patients, Hanson pin
	- Time to growth plate arrest: 34 mo
	- Signiﬁcant correction of all FAI related parameters
	- Decrease of Head Shaft Angle (Southwick): 9.0⁰
	- Decrease of the Alpha Angle: 14.5⁰
	- Signiﬁcant correlation between the reduction of the alpha-angle and age and longitudinal growth of the femoral neck
	- Klein line: increase of displacement by 1.6 mm

Femoral neck remodeling is best assessed on the frog-lateral pelvis view (Figure 4). Monitoring of the alpha-angle and the Head-Neck Offset Ratio (HNOR) are adopted by most authors to describe femoral neck remodeling and to predict the risk for FAI of the hip after SCFE.

Growth and remodeling are interconnected processes, which end with growth plate closure [6,8,45]. A longer time to physeal maturity implies a more pronounced growth and remodeling potential of the patient. This explains why post-slip FAI is less common in patients younger than 11 years [44]. The triradiate cartilage of the acetabulum is a useful predictor of the remaining growth and remodeling potential of the hip. Femoral neck growth plate fusion is expected 12 months after triradiate cartilage closure. The remaining growth and remodeling potential of the hip are significant in the presence of a wide to intermediate open triangular cartilage and may improve the cam-type deformity even in moderate slips [10]. 

The strong correlation between the residual growth and the correction of the alpha-angle supports growth-sparing slip stabilization techniques, especially in younger patients with significant remaining growth [10,44,45]. Nevertheless, the growth and remodeling-associated improvement of femoral neck deformity after in situ stabilization of SCFE are significantly lower, compared to the enormous correction obtained using the modified Dunn procedure (decrease of alpha-angle: 53⁰, a decrease of slip-angle: 43⁰) [46].

7.c. Limb Length Discrepancy after SCFE

Limb length discrepancy (LLD) after SCFE is usually the result of a shorter ipsilateral leg [21]. 

LLD in SCFE may be an apparent LLD or true LLD. True LLD is the result of the primary slip pathology (proximal migration of the femoral neck) and to the growth disturbance of the slipped physis. The surgical technique (promoting physeal arrest or growth-sparing) may also contribute to the final true LLD [1]. A mean true LLD of 14-15 mm is expected at growth plate fusion in treated moderate-to-severe SCFE [46,47]. On the other hand, apparent LLD after SCFE is also seen after moderate-to-severe slips and is the result of the limited abduction of the SCFE hip in order to avoid FAI. The patient overcomes limited hip abduction during walking by ipsilateral elevation of the pelvis on the coronal plane and simultaneous forward rotation of the pelvis on the transverse plane (movement of the pelvis precedes the movement of the thigh) [1,46]. A study reports an apparent shortening of 17 mm after a single screw fixation of SCFE [46].

Delayed diagnosis and treatment lead to greater LLD due to a slip of higher severity and less remaining growth [47]. Significant LLD with a 2 to 5 cm shorter ipsilateral limb and a 2 to 7 cm thinner ipsilateral thigh, compared to the contralateral, was found in patients after untreated SCFE [48]. On the contrary, the operated limb was only 0.5-0.8 cm shorter compared to the contralateral in patients treated for SCFE [33].

8. Is implant removal recommended?

**Figure 11:**
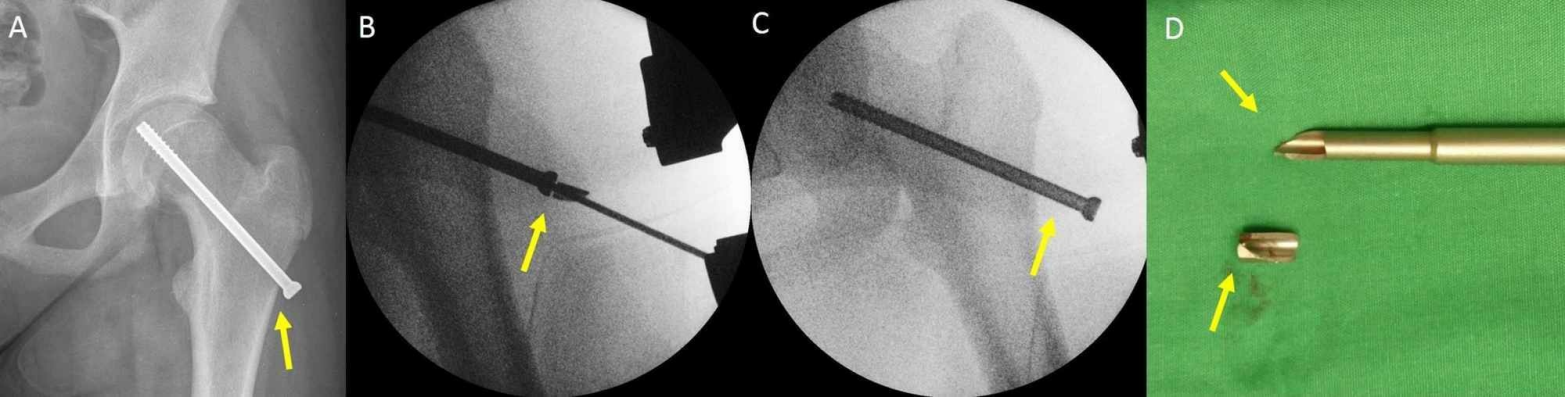
Failed removal of a 6.5mm titanium cannulated screw 18 months after insertion for a stable SCFE of the left hip of the patient in Figure 1 (A) in situ stabilization with one titanium cannulated screw (arrow), (B) incomplete unwinding of the screw, breakage of the screwdriver (arrow), (C) prominent screw, impossible reinsertion (arrow), (D) breakage of the screwdriver (arrows) SCFE: slipped capital femoral epiphysis

Implant removal after physis fusion is an additional surgical procedure, which, not infrequently (34%-50%), is accompanied by special complications [1]. A partially threaded screw may not unwind. Titanium screws bind firmly to the bone, occasionally making their removal impossible (Figure 11). Bone removal of the lateral femoral cortex to uncover and loosen an implant creates a stress raiser, which predisposes to a fracture at the region of the lesser trochanter [1]. On the other hand, there is no evidence that implants left in the bone increase the risk for late inflammation and cancer or will be an obstacle for future reconstruction surgery [1]. Implants should be removed if they cause symptoms (iliotibial band tendinitis, greater trochanter bursitis) or are loose and migrate [1]. Controversy exists whether or not to remove a well seated, asymptomatic implant. It is up to the surgeon and the patient to decide if the patient will benefit from this additional procedure.

9. Novel surgical techniques for the treatment of SCFE

Treatment of SCFE has two main goals: preventing further slippage and avoiding future FAI [18]. Growth and remodeling of the femoral neck are incapable of reversing the cam-type deformity of the femoral head-neck junction after a moderate or severe stable SCFE and protect the hip from FAI [2,6,24]. Not infrequently, FAI complicates slips of mild severity as well [1,23]. Besides, the incidence of AVN after an unstable SCFE is unacceptably high [1,11,13,18]. Novel surgical techniques aim to address these problems. 

9.a. The Role of Hip Arthroscopy in the Treatment of SCFE

In order to avoid FAI, many surgeons suggest that in situ stabilization should be combined with arthroscopic osteochondroplasty of the anterosuperior femoral neck deformity, either simultaneously with the primary procedure, or later, after physis fusion and completion of femoral neck remodeling [23,27]. Arthroscopic osteochondroplasty effectively reduces the alpha angle by 20⁰-40⁰ and increases the head-neck offset [26,27]. Early arthroscopic intervention yields better results regarding hip pain and mobility and protects the hip from permanent labral and cartilage defects [26].

Arthroscopic osteochondroplasty deals only with the femoral neck deformity after the slip. It does not restore the abnormal orientation of the femoral head after the slip (posteromedial version), relative to the weight-bearing portion of the acetabulum. Consequently, after the slip, the weight-bearing surface of the acetabulum articulates with a different portion of the femoral head instead of the original weight-bearing surface, which is covered by thicker articular cartilage. Thus even without FAI, the femoral head cartilage is subjected to abnormal loads, which could harm the cartilage in the long term. Therefore, theoretically, a modified Dunn procedure is superior to arthroscopic osteochondroplasty to prevent early hip degeneration [1].

9.b. The Modified Dunn Procedure

Proximal femoral osteotomies (Southwick, Imhauser, etc.) are late reconstruction procedures of the hip, which deal with severe gait disturbance secondary to post-slip FAI. However, these osteotomies do not restore the original hip anatomy. Subcapital neck osteotomy with femoral neck shortening is a procedure, which can be used to restore the anatomy of the hip after SCFE and thus treat SCFE, and simultaneously prevent FAI. First described by Green (1945), later described independently and named after Dunn (1964), sub-capital osteotomy with femoral neck shortening has been used in treating both stable and unstable slips [49,50]. The main concern of the procedure is to detach the capital femoral epiphysis along with the retinacula of Weitbrecht off the femoral neck, to remove the posteroinferior callus and the physeal part of the neck, and finally, to reattach the femoral head on the femoral neck. Both authors stressed the need for femoral neck shortening in order to obtain an anatomic reduction of the epiphysis on the metaphysis without tensioning the vessels of the lateral retinaculum, which supply the femoral head. However, the originally described procedure had a high risk of avascular necrosis and was abandoned for years, until Reinhold Ganz in 2001 proposed a modification of the technique, named surgical hip dislocation (SHD) [23]. SHD includes the dislocation of the femoral head (along with the retinacula of Weitbrecht) after cutting the ligamentum teres [23].

The modified Dunn procedure is an attractive option for the treatment of moderate and severe slips, provided that the physis is open [2,15,18,23,24]. Studies report less risk of AVN when the modified Dunn procedure is used (0-26%) [2]. An increasing number of studies support this method to treat SCFE and avoid FAI; however, it is not widely adopted due to its technical difficulty [15,18,23]. It has been proposed that unstable SCFE, which is complicated by AVN in up to 50% of cases, could be an indication for primary treatment using the modified Dunn procedure [11,18].

## Conclusions

Several factors affect the outcomes of slipped capital femoral epiphysis (SCFE). Except for devastating complications, such as avascular necrosis of the femoral head and chondrolysis of the hip joint, the most critical factors that must be controlled in order to obtain better results are an early diagnosis of SCFE and prevention of femoroacetabular impingement (FAI). Early diagnosis and treatment lead to less severe post-slip deformity of the femoral neck. Growth and remodeling of the hip may improve the post-slip deformity by correcting the alpha-angle and the femoral head-neck offset and decrease the risk of FAI and early hip osteoarthritis, especially in case of mild or moderate slips. In case FAI cannot be avoided through the remaining growth and remodeling potential of the hip, the restoration of the FAI-associated neck deformity by surgical means is imperative. There is increasing literature support for arthroscopic osteochondroplasty of the femoral neck deformity after stabilization of mild slips and for primary treatment of severe slips using the modified Dunn procedure. Moderate slips may benefit from both procedures. However, in situ growth-preserving one-screw fixation is currently considered the treatment of choice for SCFE, because it provides slip stability with a low risk for premature physis closure and implant-related complications. The frog lateral projection of the pelvis should always be requested when examining a non-traumatic limping adolescent.

## References

[1] Samelis PV, Papagrigorakis E (2018). Slipped capital femoral epiphysis: surgical techniques, complications, special topics. Acta Orthop Traumatol Hellen.

[2] Peck K, Herrera-Soto J (2014). Slipped capital femoral epiphysis: what's new?. Orthop Clin North Am.

[3] Guzzanti V, Falciglia F, Stanitski CL (2004). Slipped capital femoral epiphysis in skeletally immature patients. J Bone Joint Surg Br.

[4] Druschel C, Placzek R, Funk JF (2013). Growth and deformity after in situ fixation of slipped capital femoral epiphysis. Z Orthop Unfall.

[5] Wölfle-Roos JV, Urlaub S, Reichel H, Taurman R (2016). Significantly lower femoral neck growth in screw fixation of the asymptomatic contralateral hip in unilateral slipped capital femoral epiphysis. J Pediatr Orthop B.

[6] Jones JR, Paterson DC, Hillier TM, Foster BK (1990). Remodelling after pinning for slipped capital femoral epiphysis. J Bone Joint Surg Br.

[7] Roaten J, Spence DD (2016). Complications related to the treatment of slipped capital femoral epiphysis. Orthop Clin N Am.

[8] Kumm DA, Lee SH, Hackenbroch MH, Rutt J (2001). Slipped capital femoral epiphysis: a prospective study of dynamic screw ﬁxation. Clin Orthop Relat Res.

[9] Holmdahl P, Backteman T, Danielsson A, Kärrholm J, Riad J (2016). Continued growth after fixation of slipped capital femoral epiphysis. J Child Orthop.

[10] Schumann E, Zajonz D, Wojan M (2016). Treatment of chronic slipped capital femoral epiphysis: Use of dynamic epiphyseal telescopic screws. Orthopade.

[11] Loder RT (2013). What is the cause of avascular necrosis in unstable slipped capital femoral epiphysis and what can be done to lower the rate?. J Pediatr Orthop.

[12] Parikh AK, Washington ER, Bobbey AJ, Spottswood SE (2018). Evaluation of femoral head viability via bone scintigraphy in the postoperative pediatric patient. Pediatr Radiol.

[13] Riley PM, Weiner DS, Gillespie R, Weiner SD (1990). Hazards of internal fixation in the treatment of slipped capital femoral epiphysis. J Bone Joint Surg Am.

[14] Herrera-Soto JA, Duffy MF, Birnbaum MA, Vander Have KL (2008). Increased intracapsular pressures after unstable slipped capital femoral epiphysis. J Pediatr Orthop.

[15] Ziebarth K, Leunig M, Slongo T, Kim YJ, Ganz R (2013). Slipped capital femoral epiphysis: relevant pathophysiological findings with open surgery. Clin Orthop Relat Res.

[16] Sankar N, Vanderhave KL, Matheney T, Herrera-Soto JA, Karlen JW (2013). The modified Dunn procedure for unstable slipped capital femoral epiphysis. A multicenter perspective. J Bone Joint Surg Am.

[17] Wensaas A, Svenningsen S, Terjesen T (2011). Long-term outcome of slipped capital femoral epiphysis: a 38-year follow-up of 66 patients. J Child Orthop.

[18] Novais EN, Hill MK, Carry PM, Heare TC, Sink EL (2015). Modified Dunn procedure is superior to in situ pinning for short-term clinical and radiographic improvement in severe stable SCFE. Clin Orthop Relat Res.

[19] Kim YJ, Sierra RJ (2012). Report of breakout session: slipped capital femoral epiphysis management 2011. Clin Orthop Relat Res.

[20] Murgier J, de Gauzy JS, Jabbour FC, Iniguez XB, Cavaignac E, Pailhé R, Accadbled F (2014). Long-term evolution of slipped capital femoral epiphysis treated by in situ fixation: a 26 years follow-up of 11 hips. Orthop Rev (Pavia).

[21] Dodds MK, McCormack D, Mulhall KJ (2009). Femoroacetabular impingement after slipped capital femoral epiphysis: does slip severity predict clinical symptoms?. J Pediatr Orthop.

[22] Rab GT (1999). The geometry of slipped capital femoral epiphysis: implications for movement, impingement, and corrective osteotomy. J Pediatr Orthop.

[23] Leunig M, Manner HM, Turchetto L, Ganz R (2017). Femoral and acetabular re-alignment in slipped capital femoral epiphysis. J Child Orthop.

[24] Murgier J, Espié A, Bayle-Iniguez X, Cavaignac E, Chiron P (2013). Frequency of radiographic signs of slipped capital femoral epiphysiolysis sequelae in hip arthroplasty candidates for coxarthrosis. Orthop Traumatol Surg Res.

[25] Larson AN, Sierra RJ, Yu EM, Trousdale RT, Stans AA (2012). Outcomes of slipped capital femoral epiphysis treated with in situ pinning. J Pediatr Orthop.

[26] Basheer SZ, Cooper AP, Maheshwari R, Balakumar B, Madan S (2016). Arthroscopic treatment of femoroacetabular impingement following slipped capital femoral epiphysis. Bone Joint J.

[27] Tscholl P, Zingg PO, Dora C, Frey E, Dierauer S, Ramseier LE (2016). Arthroscopic osteochondroplasty in patients with mild slipped capital femoral epiphysis after in situ fixation. J Child Orthop.

[28] Wensaas A, Gunderson RB, Svenningsen S, Terjesen T (2012). Femoroacetabular impingement after slipped upper femoral epiphysis: the radiological diagnosis and clinical outcome at long-term follow-up. J Bone Joint Surg Br.

[29] Engesæter LB, Engesæter IØ, Fenstad AM (2012). Low revision rate after total hip arthroplasty in patients with pediatric hip diseases. Acta Orthop.

[30] Murray RO (1965). The aetiology of primary osteoarthritis of the hip. Br J Radiol.

[31] Clohisy JC, Dobson MA, Robison JF (2011). Radiographic structural abnormalities associated with premature, natural hip-joint failure. J Bone Joint Surg Am.

[32] Jerre R, Billing L, Hansson G, Karlsson J, Wallin J (1996). Bilaterality in slipped capital femoral epiphysis: importance of a reliable radiographic method. J Pediatr Orthop B.

[33] Hägglund G, Hannson LI, Sandström S (1987). Slipped capital femoral epiphysis in southern Sweden. Long-term results after nailing/pinning. Clin Orthop Relat Res.

[34] Swarup I, Goodbody C, Goto R, Sankar WN, Fabricant PD (2019). Risk factors for contralateral slipped capital femoral epiphysis: a meta-analysis of cohort and case-control studies. J Pediatr Orthop.

[35] Castro FP Jr, Bennett JT, Doulens K (2000). Epidemiological perspective on prophylactic pinning in patients with unilateral slipped capital femoral epiphysis. J Pediatr Orthop.

[36] Boyle MJ, Lirola JF, Hogue GD, Yen YM, Millis MB, Kim YJ (2016). The alpha angle as a predictor of contralateral slipped capital femoral epiphysis. J Child Orthop.

[37] Bhatia NN, Pirpiris M, Otsuka NY (2006). Body mass index in patients with slipped capital femoral epiphysis. J Pediatr Orthop.

[38] Lehmann TG, Engesæter IØ, Laborie LB, Lie SA, Rosendahl K, Engesæter LB (2013). Radiological findings that may indicate a prior silent slipped capital femoral epiphysis in a cohort of 2072 young adults. Bone Joint J.

[39] Albers CE, Steppacher SD, Haefeli PC, Werlen S, Hanke MS, Siebenrock KA, Tannast M (2015). Twelve percent of hips with a primary cam deformity exhibit a slip-like morphology resembling sequelae of slipped capital femoral epiphysis. Clin Orthop Relat Res.

[40] Cowell HR (1966). The significance of early diagnosis and treatment of slipping of the capital femoral epiphysis. Clin Orthop.

[41] Schur MD, Andras LM, Broom AM (2016). Continuing delay in the diagnosis of slipped capital femoral epiphysis. J Pediatr.

[42] Hesper T, Zilkens C, Bittersohl B, Krauspe R (2017). Imaging modalities in patients with slipped capital femoral epiphysis. J Child Orthop.

[43] Kallio PE, Lequesne GW, Paterson DC, Foster BK, Jones JR (1991). Ultrasonography in slipped capital femoral epiphysis. Diagnosis and assessment of severity. J Bone Joint Surg Br.

[44] Akiyama M, Nakashima Y, Kitano T (2013). Remodelling of femoral head-neck junction in slipped capital femoral epiphysis: a multicentre study. Int Orthop.

[45] Örtegren J, Björklund-Sand L, Engbom M, Tiderius CJ (2018). Continued growth of the femoral neck leads to improved remodeling after in situ fixation of slipped capital femoral epiphysis. J Pediatr Orthop.

[46] Sangeux M, Passmore E, Gomez G, Balakumar J, Graham HK (2014). Slipped capital femoral epiphysis, fixation by single screw in situ: A kinematic and radiographic study. Clin Biomech (Bristol, Avon).

[47] Kim SJ, Bloom T, Sabharwal S (2013). Leg length discrepancy in patients with slipped capital femoral epiphysis. Acta Orthop.

[48] Ordeberg G, Hansson LI, Sandström S (1984). Slipped capital femoral epiphysis in southern Sweden. Long-term result with no treatment or symptomatic primary treatment. Clin Orthop Relat Res.

[49] Green WT (1945). Slipping of the upper femoral epiphysis. Diagnostic and therapeutic considerations. Arch Surg.

[50] Dunn DM (1964). The treatment of adolescent slipping of the upper femoral epiphysis. J Bone Joint Surg Br.

